# A rare case of renal oncocytoma associated with erythrocytosis: case report

**DOI:** 10.1186/1471-2490-6-26

**Published:** 2006-09-23

**Authors:** Demetrios Radopoulos, Konstantinos Tzakas, Anastasios Tahmatzopoulos

**Affiliations:** 1 Aristotle University of Thessaloniki, 1st Department of Urology, Ethnikis Amynis 41, 54635 Thessaloniki, Greece

## Abstract

**Background:**

Oncocytomas are benign tumors of the kidney that are usually diagnosed postoperatively due to differential diagnostic problems from renal cell carcinoma. Although the latter are neoplasms that have been associated with erythrocytosis in 3.5% of cases, there are no reports in the literature about a similar occurrence in oncocytomas.

**Case presentation:**

In this case report we present a unique case of a right lower pole oncocytoma associated with erythrocytosis. Erythrocytosis subsided after partial nephrectomy.

**Conclusion:**

Erythrocytosis can sometimes occur in association with renal oncocytomas.

## Background

Under normal circumstances, erythropoietin is produced in the kidney along with a variety of other substances like renin and 1,25 dihydroxycholecalciferol. Paraneoplastic syndromes such as hypertension, anemia, pyrexia and hypercalcemia are described in up to 20% of patients with renal cell carcinomas, and most of them subside after radical nephrectomy. Erythrocytosis occurs as a paraneoplastic syndrome in 3.5% of patients with renal cell carcinoma. Polycythemia is usually attributed to pathologic erythropoietin production by the tumor or by the adjacent normal parenchyma in response to hypoxia induced by tumor growth [1]. Although cases of concomitant polycythemia have been reported for a variety of urologic tumors, there are no reports about a possible association with renal oncocytomas. In this case report we present a unique case of renal oncocytoma associated with polycythemia.

## Case presentation

A 41-year-old man presented in March 2005 to the internist due to frequent headaches and plethoric face. The CBC results showed a hematocrit of 65.1%. The number of red cells was elevated: 7,32 × 10^6^/mm^3^. The mean corpuscular volume (MCV) was within normal levels: 88,9 fl. Oxygen saturation was 99%. The patient was a non-smoker. Hemoglobin was 21.7 g/dl, with all other hematological, biochemical and urine examinations being normal, apart from a small cholesterol (232 mg/dl) and GPT (72 U/L) level elevation. The patient was taking no medications and his medical and surgical history was clear.

Findings of the abdominal ultrasound examination were normal, apart from a solid mass on the lower pole of the right kidney, measuring 8.1 × 6.1 cm, with calcifications. The left kidney was normal. On subsequent CT and MRI examination this mass was enhancing. It measured 6 × 6 × 7 cm and the differential diagnosis included oncocytoma and renal cell carcinoma. There was evidence of a tumor pseudocapsule with similar uptake to that of the normal renal parenchyma and a central part showing homogeneously diminished contrast uptake. No central scar was detected. Furthermore, the mass appeared to distort the lower pole calyces of the right kidney. No renal vein invasion or lymph node enlargement was noted. Erythropoietin level was 17.4 mIU/ml (normal range: 9–26 mIU/ml).

Subsequently, after phlebotomy (twice) to prevent perioperative thrombotic complications, the patient was subjected to open partial nephrectomy via a flank approach. This decision was based on the fact that the patient was young and had a relatively higher probability to develop chronic renal insufficiency had he been subjected to radical nephrectomy. Another reason was the favorable peripheral location of the tumor and some recent evidence that tumors>4 cm can be treated by partial nephrectomy [2]. Macroscopically, the tumor demonstrated a brownish color. It was solid, well circumscribed and showed no areas of necrosis. A central scar was apparent on macroscopic examination. Microscopically, the tumor showed the morphological and immunohistochemical characters of oncocytoma (vimentin (-), CK7 (-), CD10 (+)).

The hematocrit levels dropped to normal levels in the perioperative period (probably due to phlebotomy and blood loss during the operation). Because of the need to closely follow his postoperative status, the patient was discharged from the hospital on the 11^th ^postoperative day after an uneventful recovery period. On follow-up examination 1, 3 and 6 months later the hematocrit remained normal (42 to 43%, hemoglobin 14–14.5 g/dl) and after 9 months the hematocrit was 42.5%. Follow-up MRI at 9 months was normal and erythropoietin levels dropped to 14.5 mIU/ml one year after the operation.

## Conclusion

About 7% of surgically excised renal neoplasms are oncocytomas [3]. Most are asymptomatic at presentation and are discovered incidentally during evaluation for nonurological problems, whereas hematuria and pain occur in a minority of patients. Radiologically, a central scar is often found on ultrasound and CT, however, this is considered nonspecific and occurs in only 33% of oncocytomas [3]. Because of the lack of pathognomonic radiographic signs, the diagnosis of oncocytoma is rarely made without operative exploration.

Paraneoplastic phenomena have not been reported for renal oncocytomas. However, paraneoplastic manifestations are present in up to 20% of renal cell carcinomas, sometimes being the first clinical presentation, and most of them subside after surgical treatment of the tumor [4]. Erythropoietin has been immunolocalized to the cytoplasm of renal cell carcinoma cells in the majority of cases with clinical erythrocytosis [5]. In a study of 165 renal cell carcinomas by Ljungberg *et al*, 33% of renal cell carcinomas had elevated serum erythropoietin, however, no correlation between erythrocytosis and elevated serum erythropoietin was found [6]. From a clinical point this probably means that erythropoietin is not the sole factor responsible for erythrocytosis in those patients, as was the case in this patient.

This is the first report of an oncocytoma with associated erythrocytosis. In general, erythrocytosis can have various causes like smoking, living in high altitude, sleep apnea, chronic lung disease, hemoglobin mutations or renal disease, which were excluded during diagnostic workup. The serum erythropoietin levels were within the normal range in this case and dropped by 17% one year after the operation, in parallel with stabilisation of the hematocrit to normal levels. The possibility that erythrocytosis was a result of the response to tumor-induced hypoxia can not be excluded. The contribution of erythropoietin to erythrocytosis in this particular case is unclear, however, the fact that the erythrocytosis was corrected after tumor resection strongly suggests that this was a tumor-related occurence.

## Competing interests

The author(s) declare that they have no competing interests.

## Authors' contributions

DR conceived of the case report, performed the operation and drafted the manuscript. KT was involved in postoperative follow-up. AT was involved in postoperative follow-up and drafted the manuscript. All authors read and approved the final manuscript.

## Pre-publication history

The pre-publication history for this paper can be accessed here:



## Figures and Tables

**Figure 1:**
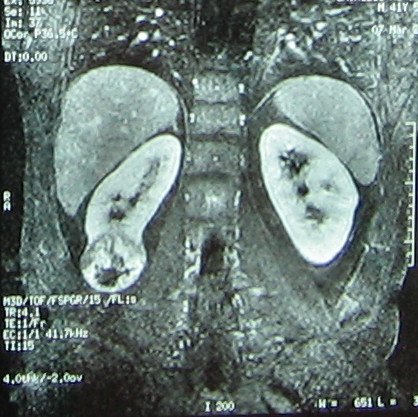
**Preoperative MRI**. MRI showing a solid right lower pole mass, well circumscribed with inhomogeneous internal structure.

**Figure 2:**
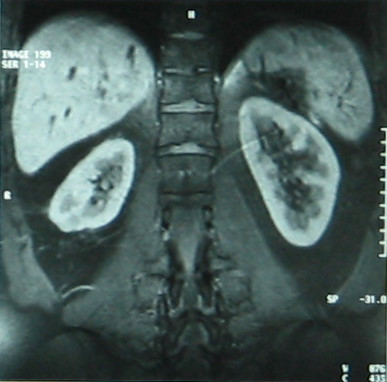
**Postoperative MRI**. Follow-up MRI at 9 months postoperatively showing normal remaining kidney with scar formation.
